# Preparation, Characterization, and Determination of Immunological Activities of Transfer Factor Specific to Human Sperm Antigen

**DOI:** 10.1155/2013/126923

**Published:** 2012-12-27

**Authors:** Jianwei Zhou, Cui Kong, Zhaohong Yuan, Junmin Luo, Rui Ma, Jiang Yu, Jinghe Cao

**Affiliations:** ^1^Clinical Laboratory, The Affiliated Hospital of Jining Medical College, Shandong, Jining 272029, China; ^2^Department of Immunology, Zunyi Medical College, Guizhou, Zunyi 563003, China; ^3^Medical Reproduction Centre, The Affiliated Hospital of Jining Medical College, Shandong, Jining 272029, China

## Abstract

*Objective.* The objective of this study was to prepare, characterize, and determine immunological activities of specific transfer factor (STF) specific to human sperm antigen (HSA) for the preparation of antisperm contraceptive vaccine that can be used as an immunocontraceptive. *Methods.* HSA-STF was prepared using the spleens of rabbits vaccinated with HSA. The specific immunological activities were examined by lymphocyte proliferation test (LPT), leukocyte adhesion inhibition test (LAIT), and by determining the concentrations of IL-4, **γ**-IFN, and IL-21. HSA-STF was a helveolous substance, having a pH value of 7.0 ± 0.4 and UV absorption maxima at 258 ± 6 nm. It contained seventeen amino acids; glycine and glutamic acids were the highest in terms of concentrations (38.8 **μ**g/mL and 36.3 **μ**g/mL, resp.). *Results.* The concentration of polypeptide was 2.34 ± 0.31 mg/mL, and ribose was 0.717 ± 0.043 mg/mL. The stimulation index for lymphocyte proliferation test was 1.84, and the leukocyte adhesion inhibition rate was 37.7%. There was a statistically significant difference between the cultural lymphocytes with HSA-STF and non-HSA-STF for **γ**-IFN and IL-21 (*P* < 0.05), but there was no statistical significance for IL-4 (*P* > 0.05). *Conclusion.* HSA-STF was prepared and characterized successfully. It had immunological activity which could transfer the immune response specific to HSA and prove to be a potential candidate for the development of male immunocontraceptive agents.

## 1. Introduction

The burgeoning population has major implications worldwide. In the face of this problem, birth control was regarded as important by many countries. Contraception is the key measure for birth control, but traditional measures have been misused in many ways; for example, easy desquamation of intrauterine ring, possible injury to person with hypodesmus, side effects of contraceptive, and condom effects. Currently available contraceptive methods are not well suited to the religious, social, economic, or health circumstances worldwide. So, there is an urgent need to develop harmless, retrievable products for contraception. Reproductive science has provided a range of fertility control measures for women, but the choices for men are few and currently limited to condoms and vasectomy [1]. Various health organizations and pharmaceutical companies actively continue to pursue research towards new contraceptive approaches [2]. Presently, based on the variable antigens (Ags) existing in the male reproductive system, researchers have developed many antibodies (Abs) or vaccines for contraception, such as Abs to SP-10 expressed in the testis that could interfere with the union of sperm and ovum [3]. The sperm-specific protein, Izumo, a member of immunoglobulin superfamily (IgSF) which is located on the sperm stimulates hamster to secrete high titer of Abs both in the genital tract and serum and further decreases their reproductive capability [4]. Antibodies to Eppin (epididymal protease inhibitor), a newly found protein secreted by testis and epididymis, could itself act as a candidate vaccine because of its ability to regulate the liquefaction of semen and limit sperm motility [5, 6]. There are several evident shortcomings regardless of the type of vaccination, such as weak antigenicity and short persistence time. Hence, it is extremely necessary to prepare an adjuvant to stimulate the immune system and enhance the immune response to Ags, especially, to pregnancy vaccines.

Specific transfer factor (STF) is low molecular weight peptides composed of number of amino acid residues and capable of transferring immunity from one cell or individual to another. It is an immunoregulatory and immunosupportive agent with normalizing effect on abnormal immune response. It is prepared by the spleen or peripheral blood lymphocytes of the animals inoculated with a certain pathogen. The essential components of STF are polypeptides and nucleotides. One of the unique features of STF is that it does not have immunogenicity and genus specificity but the STF prepared by one genus of animals like goat, rabbit, dog, and so forth has the ability of transferring the immunological activity to other genera of animals without causing hypersensitivity. Currently, several STFs have been successfully developed; mainly for herpes simplex virus type 1 (HSV-1) [7], Epstein-Barr virus [8], and *Staphylococcus aureus* antigens [9]. Also, STFs have been used in therapy of many diseases, for example, lyme, candidiasis, and herpes simplex [10–14]. However, till date no literature has been reported about the preparation of specific transfer factor related to pregnancy vaccine.

In context with the previous work on transfer factors [15–17], the present study focused on preparation, characterization, and determination of immunological activities of transfer factor specific to human sperm antigen. We anticipate that the findings from this study would provide valuable information for developing high-efficiency vaccines for birth control in China.

## 2. Materials and Methods

### 2.1. Animals, Instruments and Reagents

Rabbits and cony pigs were bought from the Experimental Animal Centre of Shandong University, China. All the animals were utilized by the rules related to experimental animals in China. The instruments utilized for the study were ultraspectrophotometer (model number 6405, JENWAY), tissue disintegrator (Fuhua of Jiangsu), Elx-800 microplate-reader (Bio-TEK), and Hitachi 835-50 analyzer. The reagents used included phytohemagglutinin (PHA), methyl thiazolyl tetrazolium (MTT), and D ribose. All the reagents were purchased from Sigma Ltd. Cytokine ELISA kit was purchased from BD Ltd.

### 2.2. Preparation of Immunogen (HSA Solution)

Sperm samples were washed five times with sterile saline and diluted with Roswell Park Memorial Institute (RPMI) 1640 medium. After the sperm suspension concentration was adjusted to 4.0 × 10^6^ sperm cells/mL, the suspension was completely subjected to comminution by Ultrasonic Cell Crusher maintained at −4°C. The human sperm antigen (HSA) solution was thus prepared.

### 2.3. Immunization of Rabbits

For the initial immunization, the rabbits were injected subcutaneously with HSA solution mixed with equivalent volumes of complete Freund's adjuvant (CFA) and incomplete Freund's adjuvant (IFA). The response to immunization with HSA was considered successful if the titer of anti-HSA was 1 : 1280. The inoculation schedule for rabbits is presented in Table 1.

### 2.4. Preparation of HSA-STF

Ten adult, male rabbits weighing 2-3 kg were bought from Experimental Animal Centre of Jining Medical College, China. The rabbits vaccinated with HSA were successfully slaughtered, and the spleens were collected after cutting off vessels and fascia. The spleens were washed with sterile saline and mechanically crushed using high-speed tissue disintegrator and, subsequently, comminuted by Ultrasonic Cell Crusher under low temperature. Following this procedure, the spleen suspension thus obtained was frozen at −20°C and thawed at 37°C. This procedure was repeated six times. The liquid was dialyzed for 48 hours using dialysis tubing having molecular weight cutoff of 5,000 dalton. The dialysate was collected after aseptic filtration and stored in a refrigerator at −20°C for further examination.

### 2.5. Analysis of General Physicochemical Properties

In accordance with the standard of transfer factor published in Chinese Pharmacopoeia (2005 Edition), the properties of HSA-STF, including color, pH, absorption peak, content of polypeptides and ribose, sterility test, pyrogen test, and safety test were determined. The content of polypeptide and ribose was detected by Orcinol assay and modified Lowry assay, respectively. Sterility test, pyrogen test, and safety test were carried out using traditional methods.

### 2.6. Analysis of Ultraviolet Spectrum and Amino Acids

The absorption peaks of HSA-STF solution were scanned in full wavelength using ultraspectrophotometer to find out the maximum absorption wavelength, and the ratio of *E*_260_/*E*_280_ was recorded. Content of amino acids in HSA-STF was detected using Hitachi 835-50 analyzer.

### 2.7. Determination of Immunological Activity

Venous blood was drawn from 20 healthy donors, and peripheral blood mononuclear cells (PBMCs) were isolated. The cells were washed twice with RPMI 1640 medium. The percentage of living cells was determined by Taipan blue staining. If the ratio was more than 95%, then, according to the experimental need, various concentrations of cell suspension were prepared with RPMI 1640 medium containing 20% fetal bovine serum (FBS). The specific immune activity was analyzed by lymphocyte proliferation test (LPT), leukocyte adhesion inhibition test (LAIT), and by detecting T-cell expansion.

#### 2.7.1. Lymphocyte Proliferation Test (LPT)

Cell suspension (100 *μ*L) with concentration 3 × 10^6^/mL was placed into seven culture wells of 96-well plate. In the first six wells, 50 *μ*L PHA, 50 *μ*L HSA with optimal concentration (4.0 × 10^6^ sperm cells/mL), and 50 *μ*L HSA-STF with gradient concentration (polypeptide concentration of HSA-STF 1 mg/mL; 0.25 mg/mL; 0.063 mg/mL; 0.016 mg/mL; 0.004 mg/mL; 0.001 mg/mL) were added. The seventh well was treated as control to which only 100 *μ*L cultural solution was added. Each well was filled with RPMI 1640 medium containing 20% FBS, until the volume was 300 *μ*L. Subsequently, the plate containing the cell suspensions and test substances were cultured at 37°C, 5% CO_2_ atmosphere for 68 hours. After the incubation period, 20 *μ*L MTT and 200 *μ*L dimethyl sulfoxide (DMSO) were, respectively, added into each well. The final solution was used for measuring the optical density (OD) by enzyme-labeled meter using dual wavelength (570 nm and 630 nm). Stimulation index (SI) was calculated using the following formula:

(1) $$\begin{array}{c} \text{SI}=\frac{{\text{OD}}_{\text{Exp}}}{{\text{OD}}_{\text{Con}}}. \end{array}$$

#### 2.7.2. Leukocyte Adhesion Inhibition Test (LAIT)

Cell suspension (100 *μ*L) with concentration 5 × 10^6^/mL was placed into the first six experimental wells of 96-well plate. Then, 50 *μ*L of HSA-STF with polypeptide concentration of 0.063 mg/mL was added. Cell suspension (100 *μ*L) plus 50 *μ*L RPMI 1640 medium containing 10% FBS was added into the successive three wells treated as controls. These solutions were cultured for 2 hours at 37°C and 5% CO_2_ atmosphere. After the incubation period, 50 *μ*L HSA with optimal concentration (4.0 × 10^6^ sperm cells/mL) and 50 *μ*L PHA solution were added to the first three experimental wells, and 50 *μ*L PHA plus RPMI 1640 medium containing 10% FBS were added to the remaining six wells. The solutions in all the wells were again cultured for 2 hours. After decanting the supernatant fraction, 180 *μ*L RPMI 1640, 20 *μ*L MTT, and 20 *μ*L DMSO were added to the remaining sperm cell fraction. The final solution was used for measuring the OD. Leukocyte adhesion inhibition rate (LAIR) was calculated using the following formula:

(2) $$\begin{array}{c} \text{LAIR}=\left[\frac{\left({\text{OD}}_{\text{Con}}-{\text{OD}}_{\text{Exp}}\right)}{{\text{OD}}_{\text{Con}}}\right]\times 100\%. \end{array}$$

#### 2.7.3. Detection of T-Cell Expansion

The procedure used for the preparation of cell culture was same as that for LAIT. The first three experimental wells were filled with 50 *μ*L HSA-STF having polypeptide concentration of 0.063 mg/mL. In the middle three wells (control 1) and the subsequent three wells (control 2), 50 *μ*L RPMI 1640 containing 10% FBS was added. These solutions were cultured for 2 hours at 37°C, 5% CO_2_ atmosphere. After the incubation period, 50 *μ*L HSA was added into the first six wells, and 50 *μ*L RPMI 1640 containing 10% FBS was added into the remaining three wells, and again cultured for 2 hours. Finally, the supernatant was carefully collected, and the concentration of IL-4, *γ*-IFN, and IL-21 was detected using ELISA kit.

#### 2.7.4. The Contents for Transfer Assays Assessed by Skin Tests

30 BALB/c mice, each weigh about 20 g, were taken as the experimental animals, and they were divided into STF, NTF (normal transfer factor, prepared by the spleen of healthy rabbits), and control group randomly, the mice of the group were given HSA-STF, NTF, and saline water, respectively, both the concentrations of HSA-STF and NTF were same as 0.63 mg/mL, and all the doses were 0.5 mL every day. On the 8th day, the pouring was stopped, and all were injected intracutaneously with HSA 0.5 mL and observed if there were any nodules formed on the skin.

### 2.8. Statistical Analysis

All data were analyzed with SPSS15.0 software. Quantitative data was presented as $\bar{x}\pm s$ (mean ± SD). Single elemental data analysis was performed using chi-square test whereas *t*-test was used for comparing paired sample sets. Statistical significance was at *P* ≤ 0.05.

## 3. Results

### 3.1. General Physicochemical Properties of HSA-STF

The HSA-STF was a helveolous substance with pH value of 7.0 ± 0.4. The HSA-STF had polypeptide content of 2.34 ± 0.31 mg/mL and ribose up to 0.717 ± 0.043 mg/mL (Table 2). In accordance with the standards of Chinese Pharmacopeia (2005 Edition), the results of protein qualitative test, sterility test, pyrogen test, and safety test were all negative. The preparation contained 17 amino acid residues in varying concentrations, out of which glycine (Gly) and glutamic acid (Glu) were the key amino acids present in the highest amount, followed by lysine (Lys) and alanine (Ala). The amounts of threonine (Thr), methionine (Met), and cystine (Cys) were the lowest (Table 3).

### 3.2. Analysis of Ultraviolet Spectrum of HSA-STF

**Figure 1:**
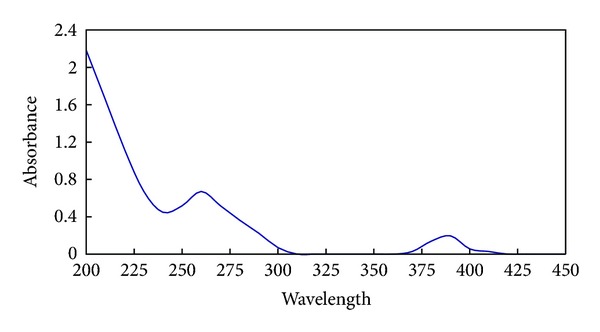
Multi wavelength scanning graph of HSA-STF.

Scanned in the range between 200 nm and 450 nm with ultraspectrophotometer, HSA-STF had a maximum absorption peak at 252~262 nm as depicted in Figure 1. The *E*_260_/*E*_280_ was 1.96 ± 0.23.

### 3.3. Lymphocyte Proliferation Test (LPT)

**Figure 2:**
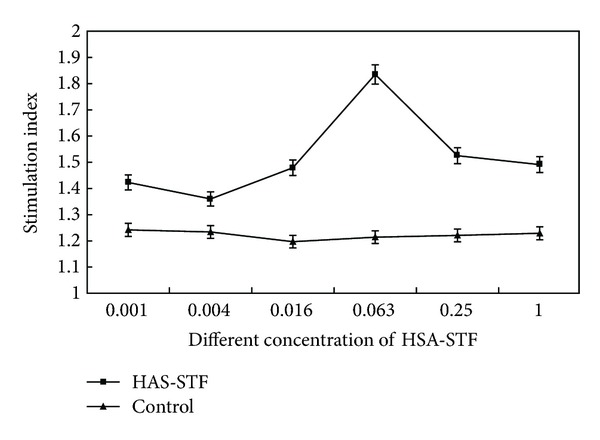
Effect of HSA-STF with different concentration of polypeptide on lymphocyte proliferation.

The proliferation of lymphocytes in the experimental wells containing HSA-STF was significant as compared to the control. HSA-STF could enhance the proliferation of lymphocytes significantly. Moreover, the effect was dependent on the concentration of HSA-STF. The stimulation index (SI) for lymphocyte proliferation test was 1.84 when the polypeptide concentration was 0.063 mg/mL (Figure 2).

### 3.4. Leukocyte Adhesion Inhibition Test (LAIT)

**Figure 3:**
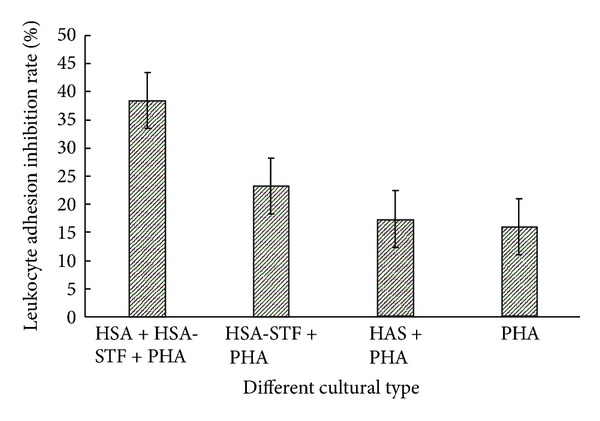
Leukocyte adhesion inhibition rate of HSA-STF.

After incubation with HSA-STF, the LAIR of the lymphocytes in the experimental wells containing HSA plus PHA was 37.7%, which was significantly higher than other wells containing either HSA or PHA (*P* < 0.05) (Figure 3). This finding suggested that HSA-STF could inhibit leukocyte adhesion, and the effect of LAI was dependent on specific antigen.

### 3.5. Expansion of T-Cell Subpopulation

Compared with the control-1 group, there was no significant difference (*P* > 0.05) in IL-4 content for the experimental group, but there was a significant difference (*P* < 0.05) between the two groups for the levels of *γ*-IFN and IL-21. Similar results were observed for the experimental group and control-2 group (Table 4).

### 3.6. The Contents for Transfer Assays Assessed by Skin Tests

In the STF group, the skin was inflamed at the site of injection, and there were marked nodules formed, while there was no nodule observed in the mice in both NTF and control groups.

## 4. Discussion

The development of vaccines based on sperm antigen is an exciting proposition and may represent a promising alternative to the currently available contraceptive methods [4]. In this study, we prepared and characterized HSA-STF successfully, and the physicochemical properties (including the color, absorption peak, the content of polypeptide, and ribose) were determined. The immunological activity of HSA-STF was also determined. The HSA-STF acted in accordance with the standard of transfer factor published in Chinese Pharmacopeia (2005 Edition). There were conflicting reports for the number of amino acids present in HSA-STF. Some reported 17 while others reported 18 amino acids [18, 19]. Moreover, the content of each amino acid varied from study to study. On the contrary, a study byKirkpatrick stated that multiple combinatorial patterns between these amino acids create a vast number of different transfer factor molecules. Such large number of molecules would then satisfy the notion that STF molecule was necessary to transfer immunity to each and every specific antigenic determinant [20].

Lymphocyte proliferation test is used to detect the nonspecific characteristic of STF. The results showed that HSA-STF stimulated the proliferation of lymphocytes, which resulted in increase in the concentration of polypeptide as increased in SI. When the concentration of polypeptide was more than 0.063 mg/mL, the SI decreased. On the contrary, this phenomenon showed, that after stimulation by HSA-STF, the increase or decrease in lymphocytes was dependent upon the concentration of polypeptide. These results were in line with another study by Lawrence [21]. A study by Xiao et al. 2004 [22] reported that the optical concentration of TF specific to tuberculosis was 0.50 mg/mL. This is probably due to the differences in HSA-STF components, which decide its stimulation activities. According to this characteristic, a study by Kirkpatrick et al. 1985 [23] was focused to prepare STF by immunizing mice with artificial amino acids. This resulted in successful transfer ofthe delayed type hypersensitivity (DTH) specific to the peptide in the unimmunized group.

In the results of LAIT, the LAIR of the group containing HSA, HSA-STF, and PHA was significantly higher than the other groups. This proved that, in the presence of HSA, transfer factor specific to HSA could significantly enhance the immunological activity of the lymphocyte. Moreover, it also raised the capability of specific response to differential immunogen. Thus, the data of this study demonstrated that HSA-STF possessed antigen specific activity, which depended on the antigen and the capacity of combining with the specific antigens [24].

Specific transfer factor plays an important role in regulating the immune response. A study by Pineda et al., 2005, [10] determined the role of STF in regulating the immune response using the mouse model of pleomorphism malignancy glioma and revealed that STF increases the number of CD2, CD4, CD8, and NK cells; it enhances the apoptosis of number of tumor cells and the expression of Th1 subpopulation in tumor tissue. Another study by Fujisawa et al. 1991 reported that, through a possible mechanism of STF, it could affect peripheral blood mononuclear cells, raise the production of leukocyte movement inhibition factor, promote the quantity of *γ*-IFN, IL-1, CD4, and CD3, and also could stimulate the expression of IL-2R [25]. The findings from a study by Fujisawa et al. 1991 [25] showed that the contents of IL-4, *γ*-IFN, and IL-21 were detected as the key products of T helper 1 cells (Th1). It also revealed that *γ*-IFN concentration was significantly higher than the lymphocytes without stimulation by HSA-STF. These results were in line with another study by Kirkpatrick et al. 1970 [26]; however, the level of IL-4 was not affected by HSA-STF. This indicated that TF specific to HSA had no effect on T helper 2 cells (Th2). These results demonstrate that HSA-STF can increase the ratio of Th1/Th2 by increasing the expression of Th1, and by relatively decreasing the Th2 clonal activity. The results of this study also demonstrate that HSA-STF could enhance cellular immunity while decreasing the humoral immunological response. Further, this study revealed that the concentration of IL-21 was the same as that of *γ*-IFN, but it was significantly higher than the lymphocytes without stimulation by HSA-STF. In line with our results [27, 28] Batten et al. (2010) and Eto et al. (2011) reported that IL-21 is a key cytokine secreted by T follicular helper cells (Tfh), it promotes the activity of Tfh and also mediates the secretion function of B cells. On the other hand, cytokines have the ability to directly affect B cells through combining IL-21R expressed on the B-cell membrane, and, thus, this helps to increase the levels of immunoglobulin secreted, which in turn enhances the humoral immunity. Thus, the results from the current study demonstrate that HSA-STF plays an important role in transferring cellular immunity to the receiver, and it also has the ability to mediate humoral immunological response.

## 5. Conclusion

In conclusion, we were successful in preparing the HSA-STF vaccine. Its physicochemical properties were evaluated, and these were in accordance with the Chinese standard of STF; HSA-STF had immunological activity which could transfer the immune response specific to HAS, and thus it could prove to be a potential candidate for the development of male immunocontraceptive agents. Also, because of its antigen specificity, HSA-STF probably has the abilities to enhance the effect of potential pregnancy vaccine specific to the HSA. Further, there is a need to conduct more robust studies to further corroborate our findings.

## Figures and Tables

**Table 1:** Schedule for inoculating rabbits with immunogen (HSA).

Week	CFA (mL)	IFA (mL)	Immunogen (mL)	Inoculation pathway
1st	0.5	0.5	1	Back, abdomen, neck, armpit
2nd	**—**	**—**	2	Back, abdomen, neck, armpit
3th	**—**	—	2	Back, abdomen, neck, armpit
4th	—	—	4	Back, abdomen, neck, armpit
5th	—	—	4	Back, abdomen, neck, armpit

“—” represents this component that was not present in the immunogenic mixture.

**Table 2:** Physicochemical properties of HSA-STF.

Tests	HSA-STF
Color	Helveolous
Protein qualitative test	Negative
Sterile test	Negative
Safety test	Negative*
Pyrogen test	Negative*
pH	7.0 ± 0.4
*E*_260_/*E*_280_	1.96 ± 0.23
Maximum absorbance	258 ± 6
Concentration of polypeptide (mg/mL)	2.34 ± 0.31
Concentration of ribose (mg/mL)	0.717 ± 0.043

*It qualifies as per Chinese Pharmacopoeia.

**Table 3:** Concentration of amino acids in HSA-STF (*μ*g/mL).

Amino acids	Concentration
Arg	11.4
Lys	33.1
Ala	22.7
Thr	3.6
Gly	38.8
Val	13.7
Ser	8.8
Pro	9.9
Ile	8.4
Leu	16.5
Met	3.4
His	7.9
Phe	11.6
Glu	36.3
Asp	7.9
Cys	2.2
Tyr	13.1

**Table 4:** Concentration of cytokines secreted by lymphocytes under the function of HSA-STF (pg/mL).

Cytokines	Experiment group	Control 1	Control 2
IL-4	234 ± 84	226 ± 51	209 ± 77
*γ*-IFN	595 ± 118	337 ± 105*	268 ± 99*
IL-21	403 ± 147	214 ± 88*	159 ± 59*

*There was a significant difference (*P* < 0.05) between experimental group and control group.

## Abbreviations

STF: Specific transfer factor
HSA: Human sperm antigen
LPT: Lymphocyte proliferation test
LAIT: Leukocyte adhesion inhibition test
IgSF: Immunoglobulin superfamily
HSV-1: Herpes simplex virus type 1
PHA: Phytohemagglutinin
MTT: Methyl thiazolyl tetrazolium.
